# The influence of climate oscillations and geological events on population differentiation of *Camponotus japonicus* in the Chinese mainland

**DOI:** 10.1002/ece3.11077

**Published:** 2024-02-22

**Authors:** Ruoqing Ma, Liangliang Zhang, Yang Xu, Cong Wei, Hong He

**Affiliations:** ^1^ Key Laboratory of National Forestry and Grassland Administration for Control of Forest Biological Disasters in Western China, College of Forestry Northwest A&F University Yangling Shaanxi China; ^2^ Key Laboratory of Plant Protection Resources and Pest Management of the Ministry of Education, College of Plant Protection Northwest A&F University Yangling Shaanxi China

**Keywords:** Chinese mainland, climate oscillations, geological events, phylogeography, social insect

## Abstract

*Camponotus japonicus* (Hymenoptera: Formicidae) is an omnivorous social insect which builds sizable colonies in sparse woodlands or cropland and spreads across multiple climatic zones in the Chinese mainland. This study aims to reveal the role of climate changes and geological events in driving the genetic structure of social insect populations by investigating the phylogenetics and historical demography of *C. japonicus* in the Chinese mainland. Phylogenetic analyses were conducted based on the mitochondria DNA dataset using MrBayes and IQ‐TREE. We constructed a haplotype network, calculated analyses of molecular variance, estimated the divergence time, and reconstructed the maximum clade credibility tree. Mismatch distribution and Bayesian skyline plots were used to infer historical population fluctuations. Additionally, ecological niche modeling was employed to predict the potential distribution of the species during the present, mid‐holocene, and last glacial maximum periods in the Chinese mainland. The phylogenetic tree and median‐joining network analyses support the presence of four distinct lineages in *C. japonicus*. These lineages exhibit significant genetic differentiation and limited gene flow. The divergence among the four lineages began in the early Pleistocene, approximately 1.41 million years ago (Ma). Subsequently, the central lineage diverged from both the northern and southern lineages around 1.16 Ma, while the northern and southern lineages diverged from each other at approximately 1.07 Ma. Population expansion was observed in the southern, central, and northern lineages prior to the last glacial maximum, while the Yunnan‐Sichuan lineage experienced a slight increase in population size in more recent times. The predicted distribution of the species corresponds well with the actual distribution. Furthermore, the current suitable habitat areas in northern Xinjiang, southern Tibet, and the southeast coastal regions have significantly decreased compared to the last glacial maximum and the mid‐holocene periods. Our results suggest that climate oscillations and geological events play an important role in driving genetic patterns and differentiation of *C. japonicus*. Mountain barriers isolate populations from each other, hinder the flow of genes, and effectively prevent the spread of this species. But at the same time, it also formed refugia at low altitudes areas such as Qinling‐Bashan Mountains and Yanshan‐Taihang Mountains and provide suitable habitats during glaciation. This study provides a good model for understanding how complex climate changes and geological events affect population genetic differentiation of social insects in the Chinese mainland.

## Graphical abstract

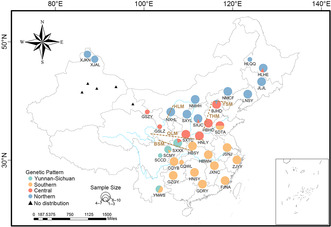

## 1 INTRODUCTION

Climatic and geographical factors play pivotal roles in influencing organism distribution and often lead to the phenomenon of allopatric speciation. Genetic variation within species populations can be attributed to underlying mechanisms such as genetic drift and constraints on gene flow (Cheng et al., 2016; Geffen et al., 2004; Hewitt, 1999). Over the past 3 million years, climate oscillations have profoundly shaped the distribution and genetic structure of numerous plant and animal species across the northern hemisphere (Hewitt, 2004). The Quaternary period witnessed several climatic oscillations in Asia, with most regions escaping ice sheet coverage (Harrison et al., 2001). While these climatic fluctuations facilitated species expansion northward, they also triggered the decline of southern populations due to the northward shift of the species' tolerance ranges during the Ice Age (Hewitt, 1999). However, this notion remains inconclusive, as previous studies were confined to North and Northeast China. Thus, comprehensive investigations encompassing widespread species throughout the Chinese mainland are imperative to address this knowledge gap.

East Asia is known for its high biodiversity, but the isolation caused by the region's mountains creates geographical barriers to the migration of many species (Liu et al., 2019; Qu et al., 2015). Geographical barriers (due to distance, mountains, water bodies, deserts, etc.) segregate populations from each other, limit gene flow, affect species distribution, and lead to allopatric speciation (Pyron & Burbrink, 2010). Although mountain systems directly prevent species transmission (mountain barrier Hypothesis), the lower elevations of mountains also provide suitable habitats during glaciation (mountain refugia Hypothesis) (Huang et al., 2010; Lei et al., 2015). Mountain barriers shape East Asian fauna by promoting speciation and maintaining high endemism (Ci et al., 2009; Huang et al., 2010). For some animals with low flight and limited migration capacity, mountains and rivers have become major inducers of population divergence, as these natural barriers geographically limit gene transmission between populations of a species (Tian et al., 2009).

The Chinese mainland encompasses an extensive and geographically diverse region, characterized by the presence of multiple climatic zones and large mountainous systems. These complex and diverse geographical conditions have played a significant role in shaping the genetic structure of biological populations (Parmesan & Yohe, 2003). The orogeny and climate change that followed the fast rise of the Qinghai‐Tibet Plateau (QTP) roughly 1.7–3.6 million years ago have led to increasing desertification in northern China, directly impacting biodiversity in the region and surrounding areas (Favre et al., 2015; Lei et al., 2015; Li & Fang, 1998; Liu et al., 2001; Raymo & Ruddiman, 1992). The rapid uplift of the Hengduan Mountains (HDM) in the southeastern QTP between the late Miocene and late Pleistocene (roughly 3.5 million years ago) has significantly altered the climate, geology, and biodiversity of the region, resulting in a higher abundance of unique species (Sun et al., 2011; Yao et al., 2010). Adjacent to the HDM, the Qinling‐Bashan Mountains (QBM) constitute a substantial mountain system spanning the east–west direction of central China. This mountain range serves as a dividing line not only between the Palearctic and Oriental realms but also between the temperate and subtropical climates of China (Fang et al., 2015; Hu et al., 2019; Huang et al., 2017). Species distributed in this area exhibit patterns of genetic differentiation in both north–south and east–west directions (Cheng et al., 2016; Yan et al., 2010; Yuan et al., 2011). In North China, the Yanshan‐Taihang Mountains represent two prominent mountainous systems, converging in the territories of Beijing and Hebei Province. Owing to their distinctive geographical attributes and climatic conditions, these areas have established conducive ecological niches for numerous plant and mammal species, rendering them as crucial distribution centers for various endemic and ecologically significant organisms (Bu et al., 2021). Southern China is located in tropical and subtropical zones, with complex topography and crisscrossing basins. Tropical and subtropical regions are never buried by ice sheets and have more stable ecosystems than temperate regions (Hewitt, 2000). Many species are widespread in southern China but intermittently distributed in this region (Lv et al., 2018). The complex geographical and climatic environment in the Chinese mainland has had an important impact on the distribution and population differentiation of plants (Zhao et al., 2013), birds (Wu et al., 2017), mammals (Li et al., 2018) and insects (Cheng et al., 2016). However, compared with a large number of studies in local regions, there is limited research across the entire Chinese mainland.

In order to expand the biogeography research of plants and animals in the Palearctic realm and Oriental realm, the appropriate species should be widespread and relatively hardy (Wahlberg & Saccheri, 2007). Among the diverse insect groups, ants stand out as one of the most common and highly evolved social insects in nature (Hölldobler & Wilson, 1990; Passera et al., 1996). There are currently 16 subfamilies, 345 genera, and 14,064 species in the world (Ant Web, 2022). Notably, many ant species are widespread and possess a remarkable cold‐hardiness, making them ideal candidates for biogeography research. For instance, Cristiano et al. (2016) conducted a study on *Acromyrmex striatus*, which revealed significant genetic diversity and habitat specificity among tropical forest ant, providing insights into its evolutionary history and neotropical biogeography. Similarly, Graham et al. (2016) investigated diversification patterns in Madagascar ants, shedding light on the evolutionary processes that shape ant populations within the unique ecological context of Madagascar, thereby contributing to a deeper understanding of the distinct biogeographic patterns on the island. Moreover, the study of ant mating behavior, particularly nuptial flight, has emerged as an essential aspect of population dispersal (Baer, 2011). After mating, newly mated dealate queens scatter on the ground to establish new colonies (Weber, 1972). Interestingly, the timing and motivation of nuptial flight exhibit species‐specific variations. For instance, *Mycetophylax simplex* queens can linger in the air for an extended period after mating and fly several kilometers to disperse the population (Cardoso et al., 2015). The object of our study, *C. japonicus*, is a widely distributed ant species in the Chinese mainland. It has a large population and strong ecological adaptability, making it an ideal biogeographic research object. After extensive sample collection in the Chinese mainland, we estimated the population structure, genetic diversity, divergence time, and historical demography of the ants, based on partial sequences of mitochondrial genes. In addition, we also modeled the potential distribution of this ant based on the MaxEnt model to predict its current and historical habitats. Our research contributes valuable insights into the biogeographic patterns and evolutionary processes across the diverse landscapes of the Chinese mainland. By conducting these studies, we aim to further our understanding of how climatic and geographical factors have shaped the distribution and differentiation of *C. japonicus* populations and shed light on the broader biogeographic patterns of social insects in the region. Moreover, the findings have important implications for the conservation of social insects and other species, aiding in designing effective strategies to preserve biodiversity in the face of ongoing environmental changes.

## 2 MATERIALS AND METHODS

### 2.1 Sampling and specimens

**FIGURE 1:**
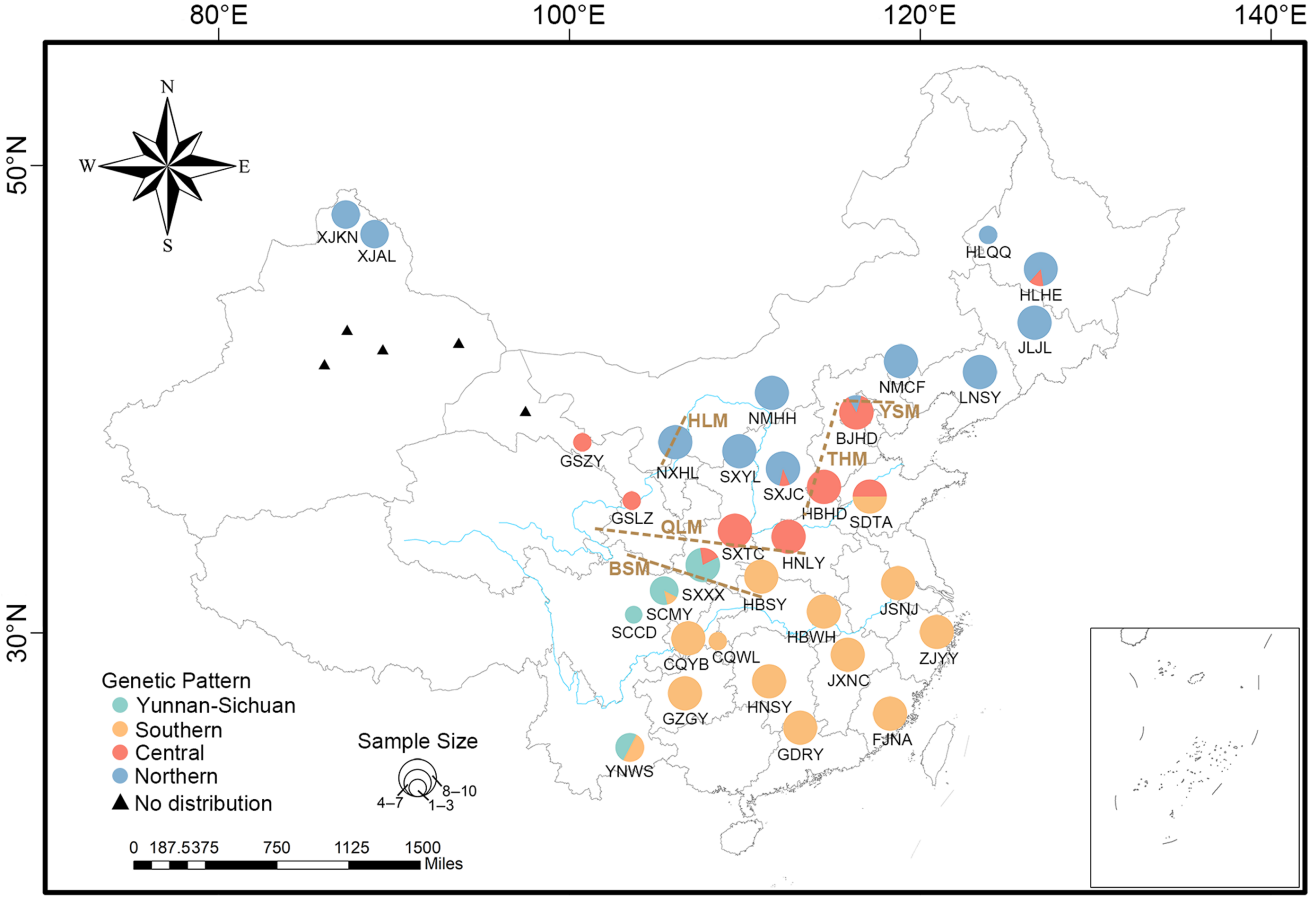
The geographic distribution of *Camponotus japonicus* sampled in this study. Four colors represent different lineages, and the size of shapes represents different sample sizes. The black triangle area in the Northwest of China is the gap in the distribution of *C. japonicus* where we did not find the species in our sampling effort. BSM, Bashan Mountains; HLM, Helan Mountains; QLM, Qinling Mountains; THM, Taihang Mountains; YSM, Yanshan Mountains.

In this study, a total of 264 colonies of *C. japonicus* were collected from 33 regions, spanning its entire distribution area across the Chinese mainland from April to November 2021 (Figure 1 and Table 1). The workers were meticulously preserved in 100% ethanol to facilitate subsequent identification and DNA extraction. Worker specimens of *C. nicobarensis* and *C. vanispinus* collected from Nanning City, Guangxi Province were selected as outgroups for phylogenetic analyses. The voucher specimens of *C. japonicus*, *C. nicobarensis*, and *C. vanispinus* were deposited at the College of Forestry, Northwest A&F University (NWAFU), situated in Yangling, China.

**TABLE 1:** Sample data for 33 populations of *Camponotus japonicus* used for molecular analysis in this study.

Code	Population	Longitude	Latitude	Date	Colonies
BJHD	Haidian, Beijing	116°36′81″	39°98′13″	Jun.2021	10
CQWL	Wulong, Chongqing	107°76′75″	29°33′21″	Nov.2021	3
CQYB	Yubei, Chongqing	106°6896″	29°61′18″	Apr.2021	7
FJNA	Nanan, Fujian	118°45′99″	25°09′36″	Apr.2021	10
GDRY	Ruyuan, Guangdong	113°23′72″	24°78′85″	Apr.2021	10
GSLZ	Lanzhou, Gansu	103°60′28″	36°05′35″	Jul.2021	3
GSZY	Zhangye, Gansu	100°43′95″	39°04′99″	Jul.2021	2
GZGY	Guiyang, Guizhou	106°69′91″	26°60′47″	Apr.2021	10
HBHD	Handan, Hebei	114°47′92″	36°63′02″	Jun.2021	10
HBSY	Shiyan, Hubei	111°07′01″	32°46′56″	Aug.2021	10
HBWH	Wuhan, Hubei	114°44′76″	30°56′46″	Aug.2021	10
HLHE	Harbin, Heilongjiang	126°72′36″	45°72′51″	Aug.2021	7
HLQQ	Qiqihaer, Heilongjiang	123°95′06″	47°34′96″	Aug.2021	3
HNLY	Luoyang, Henan	112°46′17″	34°60′94″	May.2021	10
HNSY	Shaoyang, Hunan	111°47′42″	27°23′81″	May.2021	10
JLJL	Jilin, Jilin	126°62′42″	43°88′21″	Sep.2021	10
JSNJ	Nanjing, Jiangsu	118°85′98″	32°07′61″	May.2021	9
JXNC	Nanchang, Jiangxi	115°76′57″	28°77′72″	May.2021	10
LNSY	Shenyang, Liaoning	123°43′07″	41°84′49″	Sep.2021	9
NMCF	Chifeng, Neimenggu	119°00′51″	42°29′89″	Jul.2021	10
NMHH	Huhehaote, Neimenggu	111°62′65″	40°88′59″	Jul.2021	10
NXHL	Helan Mountains, Ningxia	105°99′71″	38°72′24″	Jun.2021	8
SCMY	Mianyang, Sichuan	104°73′27″	31°48′23″	May.2021	7
SCCD	Chengdu, Sichuan	104°00′98″	30°69′22″	May.2021	3
SDTA	Taian, Shandong	117°08′32″	36°27′14″	Aug.2021	10
SXJC	Jiaocheng, Shanxi	112°14′61″	37°57′78″	May.2021	10
SXTC	Tongchuan, Shaanxi	109°08′97″	34°91′98″	May.2021	8
SXXX	Xixiang, Shaanxi	107°76′62″	32°98′47″	Apr.2021	10
SXYL	Yulin, Shaanxi	109°73′22″	38°34′17″	Apr.2021	10
XJAL	Aletai, Xinjiang	88°12′83″	47°86′88″	Jul.2021	5
XJKN	Kanasi, Xinjiang	87°17′79″	48°49′44″	Jul.2021	4
YNWS	Wenshan, Yunnan	104°11′43″	22°56′01″	Oct.2021	6
ZJYY	Yuyao, Zhejiang	121°09′33″	29°73′07″	Jun.2021	10

### 2.2 Genomic DNA extraction and PCR amplification

One hind leg of major workers was used for DNA extraction with a genomic DNA extraction kit (BioTeke, Beijing, China) following the manufacturer's protocol. Genomic DNA quality and quantity were checked using 1% agarose gel electrophoresis against a 2 kb DNA ladder marker and Thermo Scientific™ NanoDrop 2000.

Polymerase chain reaction (PCR) methods were employed to amplify fragments of Cytochrome c oxidase subunit I (*COI*), mitochondrial Cytochrome b (*Cytb*), *12S* and nuclear *28S* rRNA genes. The PCR amplification utilized primers listed in Table S1. The reaction mixture consisted of 2× Accurate Taq Master Mix (Accurate Biology, Hunan, China) at a volume of 12.5 μL, 1 μL of each primer (10 μM), 6.5 μL of DNA‐free water, and 4 μL of template DNA. The PCR cycling conditions were as follows: an initial denaturation at 94°C for 5 min, followed by 35 cycles with denaturation at 94°C for 1 min, annealing at 51.4°C, 41.9°C, 46°C for 1 min for *COI*, *Cytb*, and *12S*, respectively, extension at 72°C for 1 min, and a final extension at 72°C for 10 min. For the *28S* rRNA gene, the initial denaturation was conducted at 95°C for 3 min, followed by 37 cycles of denaturation at 95°C for 45 s, annealing at 53°C for 45 s, extension at 72°C for 1 min, and a final extension at 72°C for 7 min. PCR products were sequenced by Tsingke (Xi'an) Biotechnology Co., Ltd. All sequences have been submitted to GenBank (Accession Numbers: *C. japonicus*: *COI*: OQ266496‐OQ266759; *Cytb*: OQ288603‐OQ288868; 12*S*: OQ281023‐OQ281286; 28*S*: OQ270778‐OQ271041. *C. nicobarensis*: *COI*: OQ266760; *Cytb*: OQ288867; 12*S*: OQ281292; 28*S*: OQ271210. *C. vanispinus*: *COI*: OQ266761; *Cytb*: OQ288868; 12*S*: OQ281293; 28*S*: OQ271211).

### 2.3 Phylogenetic analyses

Chromas Pro software (Technelysium Pty Ltd., Australia) was employed to evaluate and calibrate chromatograms for each gene. Multiple sequence alignment was conducted using MAFFT v.7.0 (Katoh et al., 2019). Subsequently, sequences were manually edited and aligned using BioEdit v7.2.6.1 (Hall, 1999). Protein coding sequences were translated into amino acids using the MEGA 11.0 program (Tamura et al., 2021) to check for indels and premature stop codons. For each mtDNA marker, we chose to concatenate them for analysis. Concatenation of the individual mitochondrial DNA (mtDNA) markers was performed to integrate information from distinct markers, enabling a more comprehensive and accurate understanding of phylogenetic relationships. Prior to concatenation, we assessed the congruence among the different mtDNA markers to ensure their compatibility and consistency in terms of evolutionary relationships. Haplotypes were defined in DNASP v6.12.03 (Librado & Rozas, 2009) based on mtDNA sequence datasets. Phylogenetic analysis was carried out using PhyloSuite v.1.2.2 (Zhang et al., 2020). The best‐fit DNA substitution models were selected using ModelFinder (Kalyaanamoorthy et al., 2017). Maximum likelihood (ML) phylogenies were inferred using the IQ‐TREE software (Nguyen et al., 2015) under the HKY + F + G4 models, with the Shimodaira‐Hasegawa‐like approximate likelihood‐ratio test (SH‐aLRT) applied to assess nodal support. Additionally, 1000 ultrafast bootstraps (Minh et al., 2013) were conducted to evaluate the robustness of the inferred phylogenies. Bayesian inference was performed using MrBayes v.3.2.7a (Ronquist et al., 2012) under the HKY + F + G4 models. The Bayesian analysis involved two parallel runs of 10 million generations each, with a random tree and four Markov chains sampled every 1000 generations. Prior to constructing a consensus topology, an appropriate burn‐in was determined using Tracer v1.7 (Rambaut et al., 2018), and the initial 25% of resulting trees were discarded as burn‐out. Finally, the topology was visualized using the Figtree v.1.4.4 software (Rambaut et al., 2018).

We constructed haplotype networks using concatenated mtDNA sequences to better visualize the nonbifurcating (multifurcations and reticulations) relationships. The PopArt program was used to study the phylogeographic relationships between *C. japonicus* populations using the median‐joining method (Bandelt et al., 1999).

The estimation of temporal differentiation among haplotype lineages was conducted using the BEAST v1.10.4 software (Suchard et al., 2018), employing *COI* genes. The range of 16.2–19.9 million years ago (Ma) was adopted as the temporal reference for inferring the divergence time of *Camponotus*. Degnan et al. (2004) reported the estimation of *Camponotus* divergence within this range, which formed the basis for our selection. We employed the previously proposed ant molecular clock of the *COI* gene (0.015 per loci per million years) (Quek et al., 2004). Our BEAST analysis involved processing each XML input file, which was generated by BEAUTi, independently. We executed two independent runs for the analysis, each spanning 200 million generations and sampling every 2000 generations. Post‐run analysis was carried out using TRACER v1.7 (Rambaut et al., 2018) to assess the posterior probabilities and effective sample sizes (ESSs) of the MCMC outputs, ensuring the reliability of our Markov chain analysis. High‐confidence phylogenetic trees were extracted using TREE‐ANNOTATOR v1.8.0 (Drummond & Rambaut, 2007), allowing us to calculate 95% highest posterior density (HPD) intervals and average node times. We accounted for burn‐in by discarding the initial 25% of the data in each run. Finally, we visualized the divergence times and phylogenetic relationships using a dendrogram generated in Figtree v.1.4.4.

### 2.4 Population genetic analyses

DNASP was used to calculate the number of haplotypes (nh), haplotype diversity (hd), and nucleotide diversity (π) to assess genetic diversity. Arlequin 3.5 (Excoffier & Lischer, 2010) was used to perform analysis of molecular variance (AMOVA) and *F*_ST_ calculations with 10,000 permutations. Gene flow (*N*m) between pairs of populations was calculated based on the *F*_ST_ values using the formula: *N*m = ((1–*F*_ST_)/4*F*_ST_). To obtain correlations between genetic variation (*F*_ST_) and geographic distance (100 km), the Mantel test was performed using GenAlEx (Peakall & Smouse, 2006).

### 2.5 Demographic history

Demographic alterations in four lineages were assessed based on concatenated mtDNA genes. Tajima's *D* (Tajima, 1989) and Fu's *F*_*S*_ (Fu, 1997) statistics were computed, and 10,000 simulations were performed for each statistic to generate 95% confidence intervals and examine adherence to neutral expectations. The pairwise sequence mismatch distribution was determined using Arlequin 3.5 software with 1000 bootstrap replicates. Harpending's raggedness index (Hri) was employed to evaluate recent demographic or range expansions. To investigate population history, the Bayesian Skyline Plot (Rambaut et al., 2018) in BEAST v1.10.4 software was utilized to estimate effective population size. The Bayesian skyline joint tree prior to a piecewise linear skyline model was selected. The chain reaction spanned 200 million generations, sampling every 20,000 generations. Convergence and output of the BEAST analysis were assessed and analyzed using TRACER v1.5.

### 2.6 Historical biogeography and ecological niche modeling

In order to investigate the influence of Pleistocene climate oscillations on the geographic distribution of *C. japonicus*, a niche model was developed to assess the potential range of the species during the present (1950–2000), the mid‐holocene (MID, about 6000 years ago) and the last glacial maximum (LGM, 0.021 Ma). Records of species occurrence were sourced from scientific literature, the online database of the Global Biodiversity Information Facility (GBIF, www.gbif.org/), and the Global Ant Bioinformatics (GABI, http://globalants.org/) database, with the exception of locations that were vaguely described or exhibited unclear attributes. To mitigate the potential influence of duplicated coordinates and sampling bias, a filtering procedure was employed using the SDMTOOLBOX v1.1b software (Brown, 2014) within the ArcGIS environment. This filtering step aimed to diminish spatial autocorrelation in the species dataset.

The machine learning algorithms implemented in MaxEnt have demonstrated robust performance even with limited sample sizes (Phillips et al., 2006). Current climate data, comprising 19 bioclimatic variables at a resolution of 2.5 arc‐min, were acquired from WorldClim v. 2.0 (https://www.worldclim.org/). MID and LGM data, also at a resolution of 2.5 arc‐min, were obtained from WorldClim v. 2.0, based on the Community Climate System Model (CCSM) (Collins et al., 2006). To mitigate collinearity issues among environmental variables and their impact on parameter optimization in the MaxEnt model, multicollinearity tests were conducted using the Pearson correlation coefficient (*r*). Variables exhibiting a cross‐correlation coefficient value ≥0.8 were excluded from the analysis. As SDMs such as MaxEnt models face the challenge of balancing model complexity with goodness‐of‐fit, we employed the R package (ENMeval) to calibrate the model parameters and address these concerns (Kass et al., 2021; Warren & Seifert, 2011). The key parameters in model calibration include feature combination (FC) and regularization multiplier (RM). Based on the occurrence records and environmental variables of *C. japonicus*, the RM value was varied from 0.5 to 4, with an interval of 0.5, and six different FC combinations were evaluated: L, LQ, H, LQH, LQHP, and LQHPT (L = linear, H = hinge, Q = quadratic, P = product, and T = threshold) (Phillips & Dudík, 2008). The Akaike Information Criterion corrected (AICc) was used to assess model goodness‐of‐fit and complexity, with the minimum AICc (∆ AICc = 0) indicating the best model parameterization (Warren & Seifert, 2011). FCs were set as H, and RM was set to 1. The best MaxEnt model was replicated 10 times using the “Bootstrap” run type. The maximum number of background points was set to 10,000, the output format was specified as “Cloglog,” and the “Random Seed” option was selected to enhance model randomness, while other parameters were maintained at their default values. The accuracy of MaxEnt predictions was evaluated by employing receiver operating characteristic (ROC) analysis and calculating the corresponding area under the curve (AUC). The AUC values were categorized into four ranges: poor (.6 ≤ *p* < .7), moderate (.7 ≤ *p* < .8), good (.8 ≤ *p* < .9), and excellent (.9 ≤ *p* < 1) based on the classification scheme proposed by Swets (1988). The potential geographical distribution of *C. japonicus* was classified into four categories under current and past climates: least (*p* < .15), moderate (.15 ≤ *p* < .4), good (.4 ≤ *p* < .65), and high (.65 ≤ *p* < 1). Simultaneously, we calculated the area of suitable habitat for the current and past periods.

## 3 RESULTS

### 3.1 Phylogenetic relationships and estimation of divergence time

**FIGURE 2:**
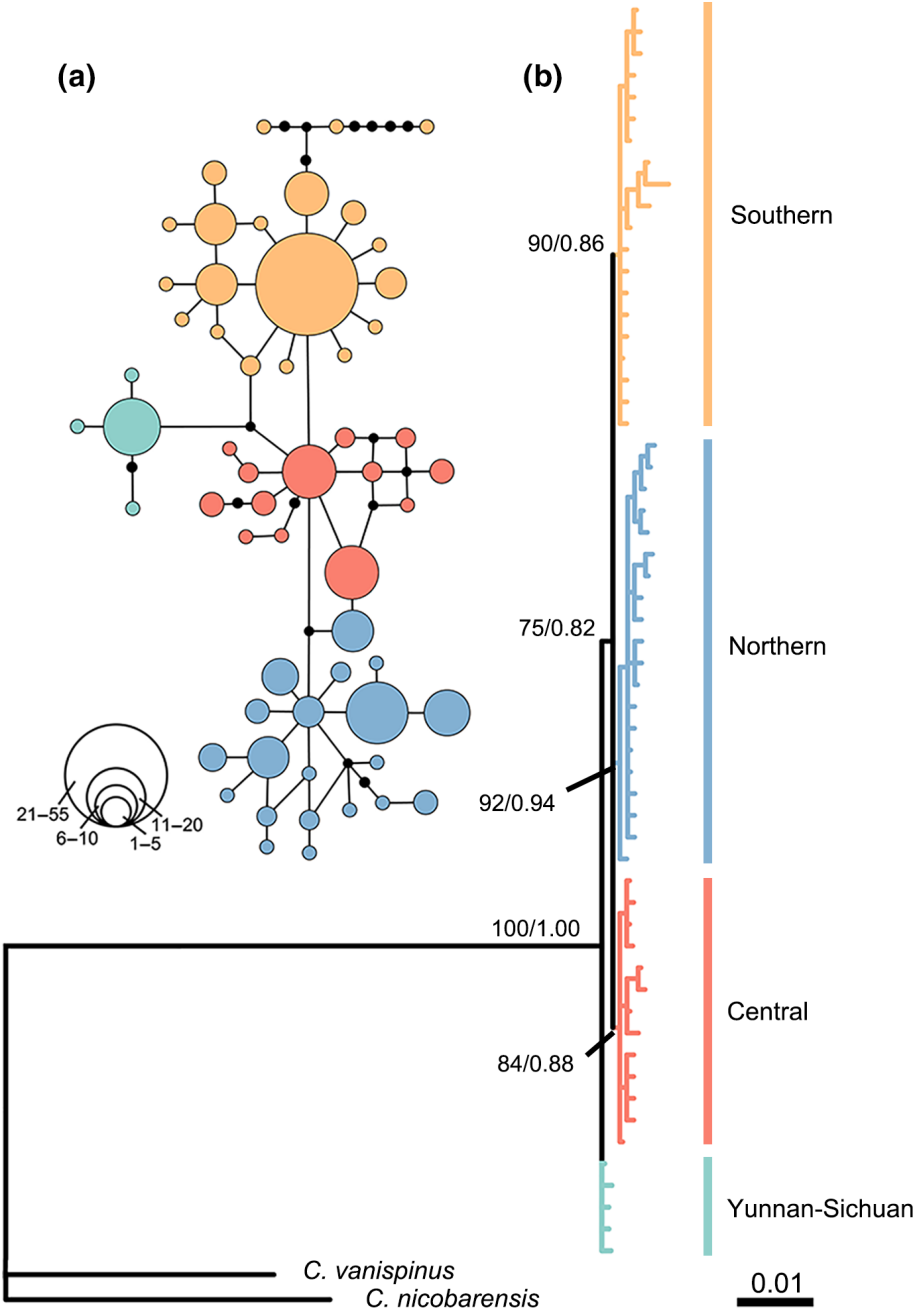
Phylogenetic tree and haplotype network profile. (a) Median‐joining network based on haplotypes for *Camponotus japonicus*. (b) Phylogram reconstructed based on haplotypes. Bayesian posterior probabilities/ML bootstrap values are indicated branches. The colors for the haplotype network correspond to the colors of four lineages in the phylogenetic tree.

We obtained 1425 bp mitochondrial genes and 677 bp nuclear genes from *C. japonicus*, including *COI* (658 bp), *Cytb* (437 bp), *12S* (332 bp) and *28S* (677 bp) genes. The analysis of polymorphic loci in the concatenated mtDNA genes revealed the presence of 57 haplotypes (Table S2). Among these, 44 haplotypes were identified as unique, indicating the existence of genetic diversity within the studied populations. Furthermore, none of the haplotypes were shared by all populations, implying distinct genetic differentiation among them. The most prevalent haplotype, H6, was shared by 55 colonies distributed in the southern region of China. Median‐joining network analysis validated the separation of the four lineages in the phylogenetic tree (Figure 2a). Loop connections between several haplotypes were observed within the northern, central, and southern lineages, suggesting frequent gene flow within each lineage.

The phylogenetic analysis of haplotypes (Figure 2b) classified all samples into four monophyletic lineages: the northern, central, southern, and Yunnan‐Sichuan lineages. The northern lineage includes haplotypes from most areas of northern China, extending to the northern foothills of the Qinling Mountains. This lineage encompasses 12 sampling sites: BJHD, HLHE, HLQQ, JLJL, LNSY, NMCF, NMHH, NXHL, SXJC, SXYL, XJAL, and XJKN. The central lineage comprises haplotypes from most areas of central China, extending to the northern Daba Mountains, and includes 10 sampling sites: BJHD, GSLZ, GSZY, HBHD, HNLY, SDTA, SXJC, SXTC, SXXX, and an additional site (HLHE) from the northern region. The southern lineage includes haplotypes from throughout southern China, encompassing 14 sampling sites: CQWL, CQYB, FJNA, GDRY, GZGY, HBSY, HBWH, HNSY, JSNJ, JXNC, SCMY, YNWS, and ZJYY, as well as one site (SDTA) from the northern region. The Yunnan‐Sichuan lineage comprises haplotypes from several mountainous areas in Southwest China, including SCCD, SCMY, SXXX, and YNWS.

**FIGURE 3:**
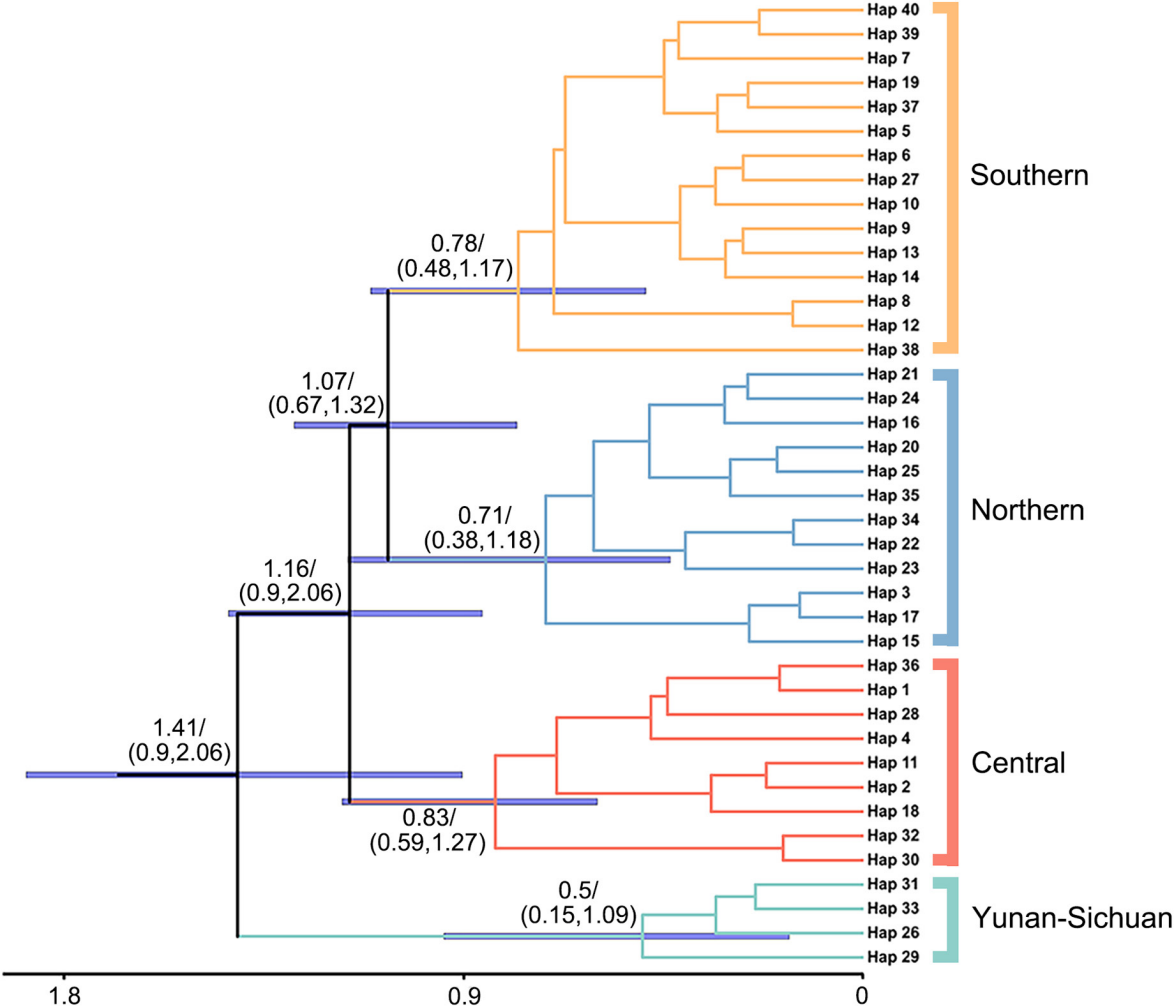
The maximum clade credibility tree from divergence‐time‐rooted phylogenetic analysis of *Camponotus japonicus* based on *COI* gene. Posterior probability support values are shown at the nodes above the branches. At the nodes, purple bars and numbers under the branches provide divergence time estimates with 95% confidence ranges.

The phylogenetic analysis of *C. japonicus* populations reveals an initial divergence event, culminating in the formation of four distinct lineages: Yunnan‐Sichuan, Central, Northern, and Southern lineages (Figure 3). The divergence of the Yunnan‐Sichuan lineage commenced during the early Pleistocene, approximately 1.41 million years ago (Ma) (95% highest posterior density (HPD): 0.9–2.06 Ma). Following this, a subsequent divergence occurred at around 1.16 Ma (95% HPD: 0.81–1.44 Ma), wherein the Central lineage diverged from the Northern and Southern lineages. The final divergence event, marking the separation of the Northern and Southern lineages, occurred at approximately 1.07 Ma (95% HPD: 0.67–1.32 Ma).

### 3.2 Genetic diversity

The northern lineage exhibited the highest haplotype diversity (hd = 0.8983) and nucleotide diversity (*π* = 0.00176) values among the four lineages, followed by the central lineage, the southern lineage, and the Yunnan‐Sichuan lineage (Table 2). The pairwise *F*_ST_ values between different lineages of *C. japonicus* ranged from 0.276 (between the central lineage and the southern lineage) to 0.584 (between the northern and the Yunnan‐Sichuan lineage), and there are no significant differences among four lineages (Table 3). Likewise, gene flow (*N*m) values varied from 0.18 (between the northern lineage and the Yunnan‐Sichuan lineage) to 0.66 (between the central lineage and the southern lineage) (Table 3). The Mantel test revealed a significant correlation (*r* = .411, *p* = .01) between genetic variation and geographic distance (Figure S1).

**TABLE 2:** Genetic diversity and neutrality tests were calculated for four lineages.

Lineages	Number of samples	Number of haplotypes	Haplotype diversity (hd)	Nucleotide diversity (π)	Tajima's *D*	Fu's *F*_*s*_
Yunnan‐Sichuan	20	4	0.2842	0.00028	−1.86788**	−2.07370**
Southern	108	20	0.7198	0.00102	−2.00062**	−13.92542**
Central	51	13	0.7867	0.00133	−1.02687	−5.02107
Northern	85	20	0.8983	0.00176	−0.99496	−8.58341

** *p* < .01.

**TABLE 3:** Pairwise genetic differentiation (*F*_ST_ in above diagonal) and gene flow (*N*_m_ in lower diagonal) between different lineages of *Camponotus japonicus*.

Lineages	Yunnan‐Sichuan	Southern	Central	Northern
Yunnan‐Sichuan	–	0.509	0.453	0.584
Southern	0.24	–	0.276	0.481
Central	0.30	0.66	–	0.415
Northern	0.18	0.27	0.35	–

The analysis of molecular variance (AMOVA) conducted on *C. japonicus* demonstrated that 55.14% (*F*_CT_ = 0.551) of the mitochondrial genetic variation occurred among the four groups (northern, central, southern, and Yunnan‐Sichuan lineages), while 23.83% (*F*_SC_ = 0.531) was attributed to variations among populations within groups, and 21.03% (*F*_ST_ = 0.790) represented variations within populations (Table 4).

**TABLE 4:** Analysis of molecular variance (AMOVA) based on concatenated mitochondrial genes.

Source of variation	df	Sum of squares	% of variance	Fixation indices	*p*‐value
Among groups	3	225	55.14	*F*_CT_ = 0.551	<.001
Among populations within groups	36	134	23.83	*F*_SC_ = 0.531	<.001
Among populations within populations	224	100	21.03	*F*_ST_ = 0.790	<.001

### 3.3 Demographic history

**FIGURE 4:**
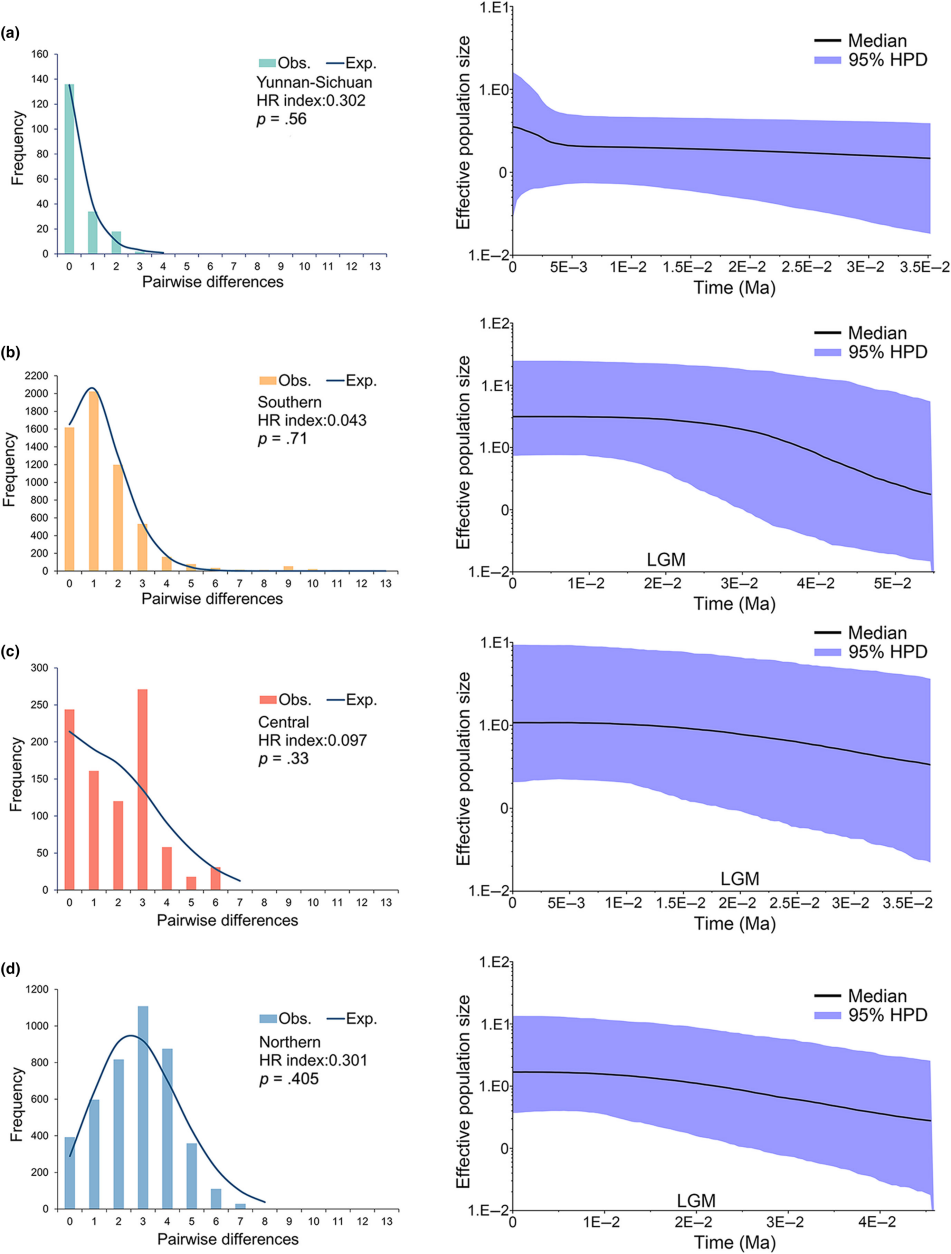
Mismatch distributions (left) and Bayesian skyline plots (right) of four lineages. (a) Yunnan‐Sichuan lineage, (b) Southern lineage, (c) Central lineage, (d) Northern lineage. The mismatch distribution is denoted by vertical bars in four colors, and the expected distribution under the population expansion model is represented by navy blue lines. Harpending's raggedness (HR) index is shown. In Bayesian skyline plots, the mean estimate is enclosed within the 95% highest posterior densities. LGM, Last glacial maximum.

Neutrality tests were performed on the combined mtDNA dataset of *C. japonicus*, revealing negative Tajima's *D* and Fu's *F*_*S*_ values in all four lineages, with the Yunnan‐Sichuan and southern lineages displaying statistically significant negative values for both statistics (Table 2). These findings provide strong evidence against the hypothesis of neutral evolution in the geographic populations of *C. japonicus*, suggesting recent population expansion in all four lineages. The results of the mismatch distribution analysis also support this conclusion, showing population expansion in all four lineages (Figure 4). Specifically, the southern, central, and northern lineages exhibited significant increases in population size before the LGM, while that of the Yunnan‐Sichuan lineage experienced a slight increase in population size in more recent time.

### 3.4 Potential distribution change

**FIGURE 5:**
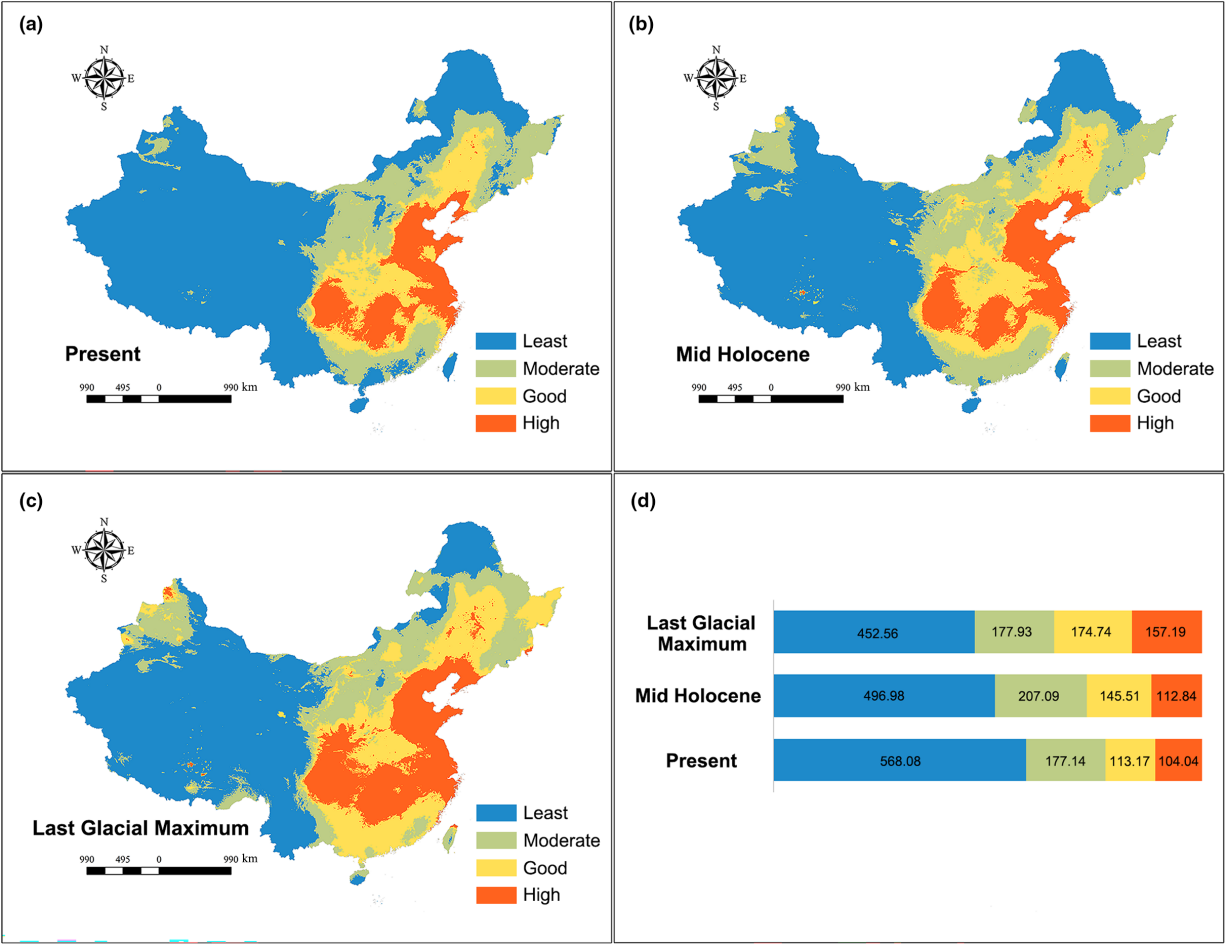
Potential distribution of the probability of occurrence for *Camponotus japonicus*. (a) Present, (b) Mid‐holocene, (c) Last glacial maximum, (d) Potential distribution area. Ecological niche models were established by the extant occurrence points. Different colors represent different predicted potentials, with the least potential in blue, moderate potential in green, good potential in yellow, and high potential in red.

The ecological niche model for *C. japonicus* demonstrates a high level of predictive accuracy, as evidenced by an area under the receiver operating characteristic curve of 0.869 (*p* < .001) (Figure S2). A final model comprising five variables was selected and retained (Table S3): minimum temperature of the coldest month (BIO06), isothermality (BIO2/BIO7) (×100) (BIO03), precipitation of the wettest month (BIO13), mean temperature of the warmest quarter (BIO10), and precipitation seasonality (coefficient of variation) (BIO15). The predicted distribution of the species under present conditions (Figure 5a) closely aligns with the observed distribution, particularly in the central, southern, and northeastern regions of the Chinese mainland. The extent of suitable habitat during the LGM exceeded that of the MID, while the MID period exhibited a larger suitable habitat area compared to the present (Figure 5d). Notably, the current suitable habitat areas in northern Xinjiang, southern Tibet, and the southeast coastal areas have experienced a significant reduction when compared to the LGM and the MID (Figure 5a–c).

## 4 DISCUSSION

In this study, we investigated the phylogenetic relationships, divergence time, and genetic diversity of *C. japonicus* populations based on mitochondrial genes. Our findings revealed the presence of four lineages (the northern, central, southern, and Yunnan‐Sichuan lineages), each exhibiting unique haplotypes and geographical distributions, indicating genetic differentiation and diversity among populations. The divergence time analysis suggests that these lineages began to diverge during the early Pleistocene, with subsequent divergence events leading to the formation of distinct lineages. The northern lineage exhibited the highest genetic diversity among the four lineages, followed by the central, southern, and Yunnan‐Sichuan lineages. Demographic history analysis provided evidence of recent population expansion in all four lineages, suggesting a dynamic population history in response to environmental changes. The potential distribution change analysis indicated shifts in suitable habitat areas over time, with significant reductions in certain regions compared to the LGM and the MID. Furthermore, the distribution pattern and genetic differentiation observed in *C. japonicus* are likely closely linked to geological events and climatic fluctuations, as well as the species' biological characteristics.

### 4.1 Influence of geological events

Our results of the molecular dating analysis have revealed intriguing correlations between lineage divergence times and major geological events. Notably, the initial divergence event in the *C. japonicus* lineage, estimated to have occurred around 1.41 million years ago (Ma), coincides with the third phase of the Qinghai‐Tibetan Plateau Uplift. This phase, characterized by the uplift of the Qinghai‐Tibet Plateau, has played a crucial role in shaping the evolutionary trajectory and environmental adaptations of this species (Li & Fang, 1998). Similarly, the divergence of the central lineage from its northern and southern counterparts, occurring at 1.16 Ma and 1.07 Ma respectively, appears to be temporally aligned with the Kunlun Yellow River Movement, which spanned from approximately 1.1 to 0.6 Ma (Li & Fang, 1998). This movement significantly raised the Qinghai‐Tibet Plateau's altitude, with an average elevation exceeding 3000 m and mountains surpassing 4000 m, thereby extending the permafrost zone across a vast area of the plateau (Shi et al., 1995). These close associations indicate that geological events have played a pivotal role in driving population differentiation and lineage formation within *C. japonicus*.

Geological processes contribute to the orogenesis of mountain ranges, which serve as elevated barriers promoting speciation and enhancing biodiversity (Cheng et al., 2016). The Qinling Mountain range, extending from the eastern periphery of the Qinghai‐Tibet Plateau to the southwestern segment of the North China Plain, constitutes a significant geographical demarcation. Within this mountain range, the northern and southern foothills exhibit pronounced distinctions in terms of habitat, climate, and species diversity, making them crucial areas for biodiversity and species origination (Lei et al., 2003). Notably, the central lineage situated in the northern region of the Qinling Mountains, along with the southern and Yunnan‐Sichuan lineages, shows conspicuous genetic differentiation, implying a pronounced north–south genetic pattern. Additionally, the Bashan Mountains, located south of the Qinling Mountains and roughly parallel to them, represent another important barrier. The populations of *C. japonicus* situated in the western regions between the two mountain ranges show genetic affinities with the central and Yunnan‐Sichuan lineages, while the population in the eastern regions exhibits close genetic relationships with the southern lineages (Figure 1). This observed distribution pattern distinctly signifies an east–west axis of genetic differentiation within the species. The species distributed in this mountain range exhibit north–south and east–west genetic differentiation patterns, consistent with the findings of this study. This distribution pattern underscores the impact of mountain barriers in shaping population structure and facilitating genetic isolation (Cheng et al., 2016; Yan et al., 2010; Yuan et al., 2011).

Furthermore, the Yanshan‐Taihang mountainous region has served as a formidable barrier, impeding the dispersal of the *C. japonicus* population and culminating in pronounced genetic differentiation between the central and northern lineages within this range. Considering the distribution ranges of the four lineages of *C. japonicus*, it is evident that the Qinling‐Bashan Mountains and the Yanshan‐Taihang Mountains are the driving factors behind the geographical genetic structure of *C. japonicus*. Moreover, low elevations in the Qinling‐Bashan Mountains and Yanshan‐Taihang Mountains have served as favorable habitats for the species during glaciation, and ecological niche simulation results further support the idea that these areas acted as refugia during challenging climatic conditions. Overall, the close associations between lineage divergence and geological events, along with the impact of mountain barriers, highlight the pivotal role of geological processes in shaping the genetic structure and distribution of *C. japonicus* populations.

### 4.2 Influence of climate oscillations

The climatic oscillations resulting from the repeated alternation of Pleistocene glaciations have exerted significant effects on the evolutionary history and distribution patterns of extant species in China (Hugall et al., 2002). These impacts encompass phenomena such as population contraction, habitat fragmentation, and instances of local extinction. Conversely, certain species adapted to colder and drier conditions have experienced population expansion during glacial events (Leite et al., 2016; Tian et al., 2010). During the LGM, the prevailing cooler global climatic conditions likely impacted the habitat dynamics of *C. japonicus*, a species characterized by its notable adaptability. These climatic conditions, characterized by lower temperatures and specific precipitation patterns, could have altered the availability and distribution of suitable habitats. As the climate warmed during the MID, and further into the modern era, these habitats likely experienced changes. In particular, the recent global warming has led to a notable reduction in suitable habitats in areas such as northern Xinjiang, southern Tibet, and the southeastern coastal regions. In the arid and semi‐arid regions of northwest China, encompassing areas such as northern Xinjiang and Gansu, observations of this ant species have been restricted to specific locales, as reported by Zhang and Zhou (2015). The habitat of *C. japonicus* in Northwest China is diminishing due to climate change and human population expansion. Surprisingly, *C. japonicus* was also discovered on Helan Mountain in Northwest China, at an elevation of 2300 m above sea level. Above this altitude, the area is occupied by *Camponotus herculeanus*. Helan Mountain is surrounded by desert or semi‐desert landscapes, which hinder the less mobile population from interacting with their counterparts outside the mountain. The diverse environmental contexts, spanning mountainous, desert, and semi‐desert ecosystems, have precipitated accelerated population differentiation (*F*_ST_ > 0.25) on Helan Mountain, potentially catalyzing novel speciation events through geographical isolation (Liu et al., 2019). This research substantially augments our comprehension of the diversity, evolutionary lineage, and environmental adaptability of *C. japonicus*.

This research significantly deepens our understanding of the diversity, evolutionary history, and environmental adaptation of *C. japonicus*, providing empirical evidence for the influence of geological events and climate oscillations on species differentiation and adaptation. This enriches our knowledge in the fields of ecology and evolutionary biogeography. However, it is important to acknowledge certain limitations. Our analysis, primarily based on mitochondrial genes, may not have captured the full spectrum of genetic variations. In our future research endeavors, we plan to address this gap by including additional polymorphic nuclear markers. Additionally, the scope of this research, limited to the Chinese mainland, might not fully represent the genetic diversity across the entire distribution of *C. japonicus*. We aim to expand the geographical scope of our study to encompass the full distribution of the species. These efforts are crucial for a more comprehensive understanding of the species' genetic structure, its evolutionary dynamics, and its response to environmental changes. By undertaking these steps, we hope to overcome the current limitations and further advance our knowledge in this critical area of study.

## AUTHOR CONTRIBUTIONS

**Ruoqing Ma:** Data curation (equal); formal analysis (equal); investigation (equal). **Liangliang Zhang:** Data curation (equal); investigation (equal). **Yang Xu:** Conceptualization (equal); methodology (equal). **Cong Wei:** Conceptualization (equal); methodology (equal). **Hong He:** Conceptualization (equal); funding acquisition (equal); methodology (equal); project administration (equal).

## CONFLICT OF INTEREST STATEMENT

The authors declare that there are no conflicts of interest regarding the publication of this paper.

## Supporting information


Appendix S1.

## Data Availability

The sequencing data were uploaded to GenBank (Accession Numbers: *C. japonicus*: COI: OQ266496‐OQ266759; Cytb: OQ288603‐OQ288868; 12S: OQ281023‐OQ281286; 28S: OQ270778‐OQ271041. *C. nicobarensis*: COI: OQ266760; Cytb: OQ288867; 12S: OQ281292; 28S: OQ271210. *C. vanispinus*: COI: OQ266761; Cytb: OQ288868; 12S: OQ281293; 28S: OQ271211).
