# Supplementary material for: The influence of climate oscillations and geological events on population differentiation of Camponotus japonicus in the Chinese mainland
Source: Ecol Evol. 2024 Feb 22;14(2):e11077. doi: 10.1002/ece3.11077 (PMC10883248; doi:10.1002/ece3.11077)
Supplement: Supplementary file 1 — Appendix S1. [file ECE3-14-e11077-s001.docx]

**Supplementary Materials:**

**Table S1.** Primers used in PCR amplifying and sequencing in this study.

| Gene fragment | Primer | Primer sequence 5’–3’ | Reference |
| --- | --- | --- | --- |
| *COI* | LCO1490 | GGTCAACAAATCATAAAGATATTGG | Folmer *et al.* |
|  | HCO2198 | TAAACTTCAGGGTGACCAAAAAATCA |  |
| *Cytb* | Cb1-fw | TATGTACTACCATGAGGACAAATATC | Jermiin & Crozier. |
|  | CB2 | ATTACACCTCCTAATTTATTAGGAAY |  |
| *12S* | 12Sma | CTGGGATTAGATACCCTGTTAT | Cook *et al.* |
|  | 12Smb | CAGAGAGTGACGGGCGATTTGT |  |
| *28S* | 28Sforward-F2 | AGAGAGAGAGTTCAAGAGTACGTG | Belshaw *et al.* |
|  | 28Sreverse-3DR | TAGTTCACCATCTTTCGGGTC |  |

**References for Table S1**

Folmer, O., Black, M., Hoeh, W., Lutz, R. & Vrijenhoek, R., (1994). DNA primers for amplification of mitochondrial cytochrome c oxidase subunit I from diverse metazoan invertebrates. *Molecular Marine Biology and Biotechnology*, **3**, 294-299.

Jermiin, L.S., Crozier, R.H., (1994). The cytochrome b region in the mitochondrial DNA of the ant *Tetraponera rufoniger*: sequence divergence in Hymenoptera may be associated with nucleotide content. *Journal of Molecular Evolution*, **38**, 282-294.

Cook, C.E., Austin, J.J. & Disney, H.L., (2004). A mitochondrial 12S and 16S rRNA phylogeny of critical genera of Phoridae (Diptera) and related families of Aschiza. Zootaxa, **593**, 1-11.

Belshaw, R., Lopez-Vaamonde, C., Degerli, N., Quicke, D. L. J. (2001). Paraphyletic taxa and taxonomic chaining: evaluation of the classification of braconine wasps (Hymenoptera: Braconidae) using 28S D2-3 rDNA sequences and morphological characters. *Biological Journal of the Linnean Society*, **73**, 411-424

**Table S2.** List of the haplotypes of *C. japonicus* with different genes.

| Population | *COI* | *COI* + *Cytb* + *12S* |
| --- | --- | --- |
| BJHD | H­1, H­2, H3, H­4 | H1, H­2, H3, H­4, H5 |
| CQWL | H5 | H6 |
| CQYB | H­5 | H­6 |
| FJNA | H5, H­6, H7 | H6, H7, H8 |
| GDRY | H­5, H8, H­9, H10 | H6, H­9, H­10, H­11, H12 |
| GSLZ | H11 | H­13 |
| GSZY | H­2 | H14 |
| GZGY | H­5 | H15 |
| HBHD | H­1, H2 | H1, H2 |
| HBSY | H5, H­8 | H6, H10, H­16 |
| HBWH | H­8, H­12, H13, H14 | H­16, H17, H18, H19, H20 |
| HLHE | H­2, H3, H15 | H2, H3, H21 |
| HLQQ | H3, H16, H­17 | H3, H22, H23 |
| HNLY | H1, H2, H18 | H­1, H2, H24, H25 |
| HNSY | H5, H8 | H6, H16 |
| JLJL | H3, H1­6 | H3, H22 |
| JSNJ | H5 | H6, H10 |
| JXNC | H5, H19 | H6, H26, H27 |
| LNSY | H3, H1­7 | H3, H23 |
| NMCF | H­3, H1­7 | H3, H23 |
| NMHH | H­20, H21, H22 | H­28, H29, H30, H­31, H32, H33 |
| NXHL | H20, H23, H24, H25 | H­34, H35, H36, H37, H38 |
| SCMY | H5, H26 | H6, H39 |
| SCCD | H26 | H39 |
| SDTA | H2, H27 | H2, H40 |
| SXJC | H2, H20, H22 | H­2, H34, H41, H42 |
| SXTC | H1, H2, H28 | H1, H2, H43, H44, H45 |
| SXXX | H26, H29, H30, H31, H32, H33 | H39, H46, H47, H48, H49, H50 |
| SXYL | H22, H34, H35 | H41, H51, H52 |
| XJAL | H36 | H53 |
| XJKN | H36 | H53 |
| YNWS | H26, H38, H39, H40 | H39, H54, H­55, H56 |
| ZJYY | H5, H37 | H6, H57 |

**Table S3.** The environmental variables contributed to the model of *Camponotus japonicus*.

| Code | Environmental Variable | Percent Contribution (%) |
| --- | --- | --- |
| BIO06 | Min Temperature of Coldest Month | 30.1 |
| BIO03 | Isothermality (BIO2/BIO7) (×100) | 26.5 |
| BIO13 | Precipitation of Wettest Month | 23.9 |
| BIO10 | Mean Temperature of Warmest Quarter | 10.0 |
| BIO15 | Precipitation Seasonality (Coefficient of Variation) | 9.5 |

**Figure S1.** The Mantel tests between genetic differentiation (*F_ST_*) and geographic distance (100 km) of *C. japonicus*.


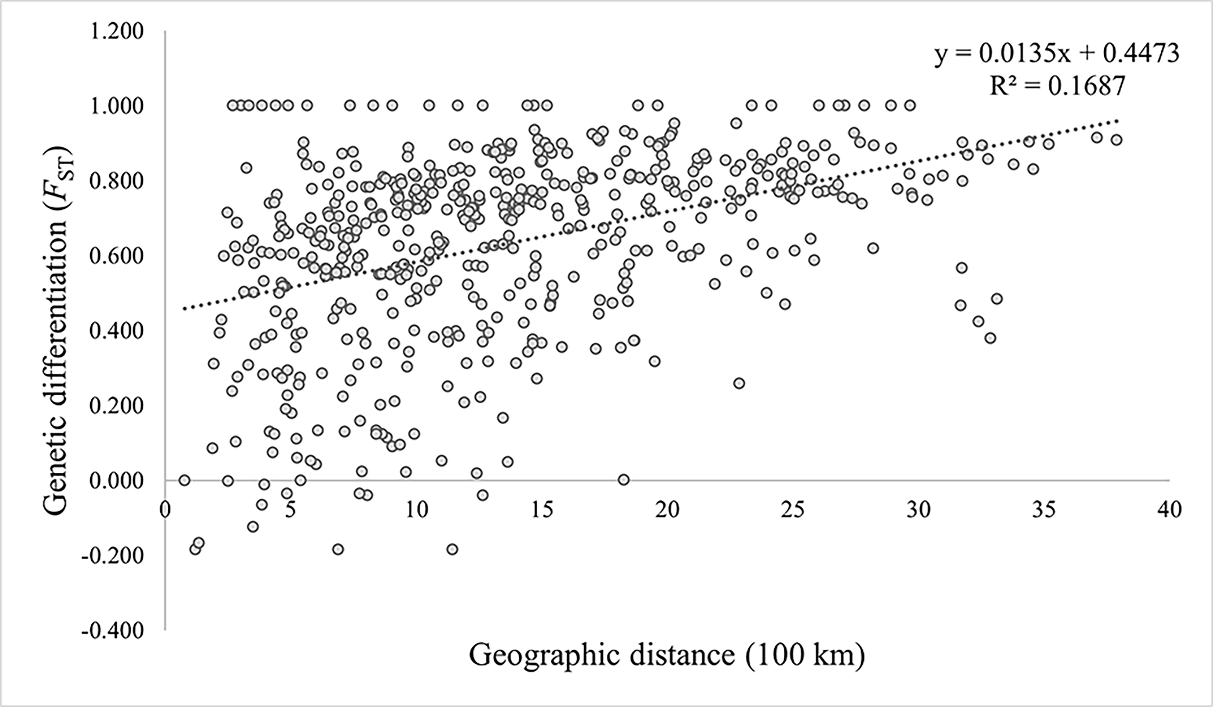


**Figure S2.** ROC curve and AUC values for the model.


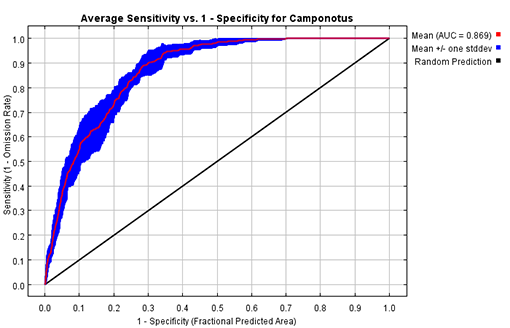


**Figure S3.** Relation between 19 environmental variables

**
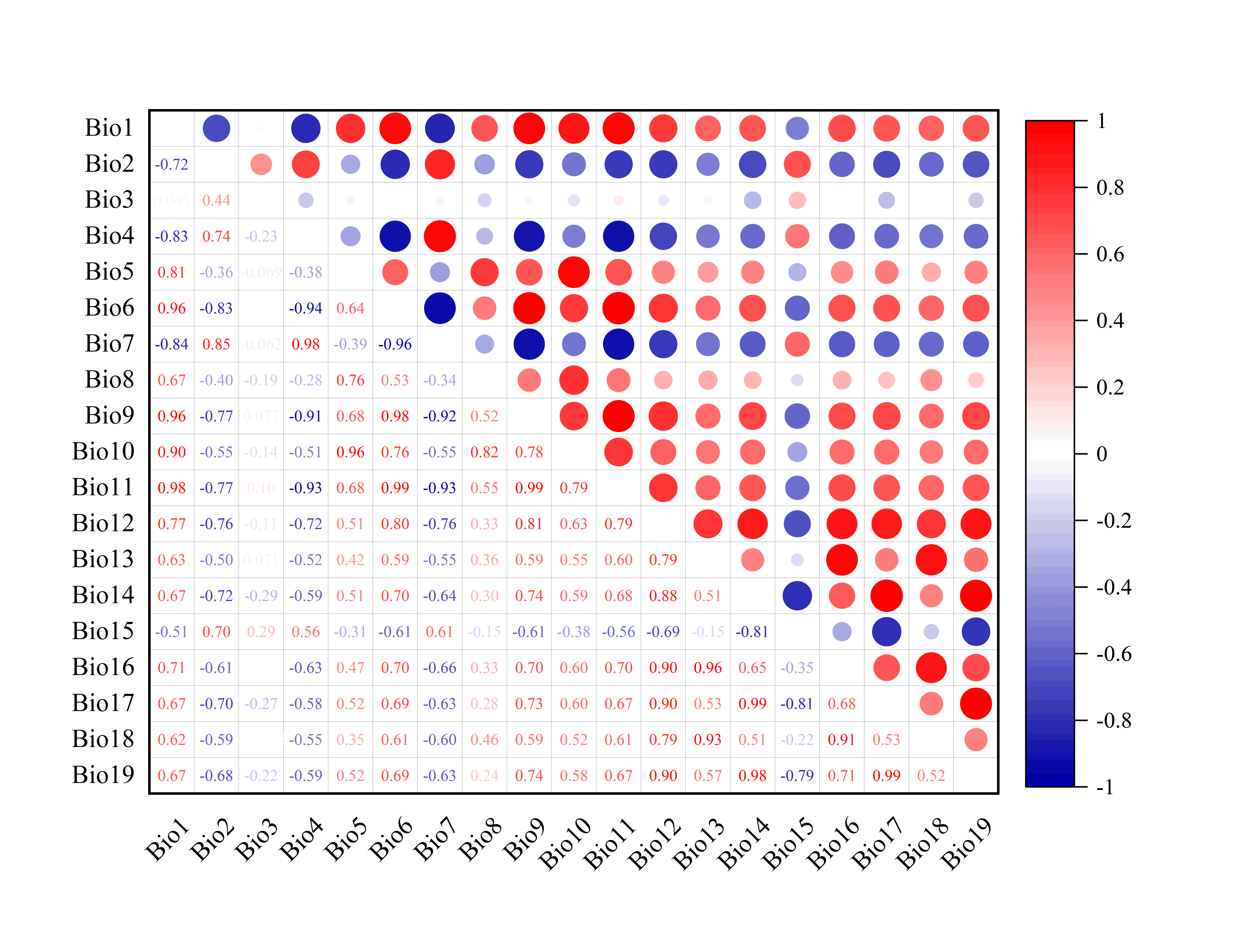
**
